# Management of blood thinning medications in elderly populations presenting with rectal bleeding: Are we doing right?

**DOI:** 10.12688/f1000research.149548.1

**Published:** 2024-06-04

**Authors:** Akshay Bavikatte, Boby Sebastian, sade Uwaoma

**Affiliations:** 1 West Suffolk NHS Foundation Trust, Bury Saint Edmunds, England, UK

**Keywords:** blood thinning medication, anticoagulants, per rectal bleed, reintiation of blood thinners

## Abstract

**Introduction:**

Rectal bleeding commonly occurs in elderly patients using blood thinners, posing management challenges due to limited guidance on reversal agents and medication restart criteria. This study aims to review the demographics and management of elderly patients with rectal bleeding while on blood thinners.

**Methods:**

A retrospective analysis of patients aged 60 or older presenting with rectal bleeding at West Suffolk Hospital’s emergency department was conducted from January 2018 to December 2020. Data were extracted from electronic records, focusing on patients using blood thinners and adhering to British Society of Gastroenterology guidelines. All patients ceased blood-thinning medications upon admission. The hospital’s ethics committee approved the study, which focused on demographics, diagnosis, and management, particularly regarding re-initiation of blood-thinning medicines.

**Results:**

During the study period, 170 patients were admitted to the emergency department of West Suffolk Hospital. 93 (54.71%) patients were included in the study. The average age of the participants was 82 years, and 62.3% were male. All patients were followed up for three months. Atrial fibrillation accounted for 52% of patients, while previous strokes accounted for 20%. The most typical pathology was diverticulosis.

Regarding restarting of anticoagulants, Among patients on DOAC (Direct oral anticoagulant), 39% were restarted on discharge, 23% were switched to warfarin, and another 23% were not restarted; 15% planned to restart after seven days. For those on Warfarin, 62% were restarted on discharge, 22% stopped the medication, and the rest were switched to Dual Oral Anticoagulant. Among aspirin patients, 60% were restarted at discharge, while the remaining discontinued. All patients receiving clopidogrel and dual antiplatelet therapy were started at discharge. None of the patients were readmitted during the follow-up period of 3 months.

**Conclusion:**

Restarting of blood-thinning drugs in patients with rectal bleeding is subject to individual patient variation. Necessitates more extensive trials to achieve greater standardization.

## Introduction

Rectal bleeding is a prevalent adverse effect in elderly patients taking blood thinners. Managing these patients can be challenging because of the scarcity of available guidance on the use of reversal agents and the decision to restart medication. ^1^ Anticoagulant therapy is a major risk factor for rectal bleeding. It is estimated that over 6 million patients in the United States are treated with anticoagulants. ^2^

The prevalence of blood thinning medications in the elderly population is a critical aspect of healthcare management. Studies have shown that the elderly often have a higher prevalence of chronic diseases, leading to the common use of multiple medications, a phenomenon known as polypharmacy, putting them at an increased risk of bleeding. ^3^ The decision to restart anticoagulation therapy following an episode of rectal bleeding is a complex process that involves the assessment of individual patient risk factors and preferences. Factors such as the underlying indication for anticoagulation, severity of the bleeding episode, and overall risk of thrombosis must be carefully considered. ^4^ Furthermore, patient demographics, such as age and comorbidities like rectal bleeding, can influence the decision-making process regarding anticoagulation therapy. ^5^

Shared decision-making and a pragmatic approach are crucial in determining the restart of anticoagulants in patients with rectal bleeding, emphasizing the importance of considering additional clinical factors, individual physician experiences, and patient preferences in decision-making and advocating for shared decision-making. ^6^

We performed a retrospective study to review patient demographics, pathology, and management of blood-thinning medications in elderly individuals admitted with rectal bleeding in our hospital and to assess the plan for restarting blood thinners.

## Methods

A retrospective analysis of individuals aged 60 years or older who presented with rectal bleeding to the West Suffolk Hospital’s emergency department between January 2018 and December 2020 was conducted. The study enrolled patients receiving anticoagulant therapy presenting with per rectal bleed. Patient care followed the protocols outlined by the British Society of Gastroenterology, ^7^ emphasizing prompt assessment, resuscitation, and endoscopic examination within 24 hours to identify the bleeding origin. Management options included endoscopic haemostasis, pharmaceutical intervention, or interventional radiology, with an emphasis on collaborative multidisciplinary care to optimize outcomes. Patients were stratified based on stability, with unstable cases undergoing post-resuscitation CT angiography for bleeding localization, while stable patients were admitted for observation. Bleeding etiology was determined during the same admission through gastroscopy and flexible sigmoidoscopy and all patients discontinued their blood thinning medications upon admission. The study focused on patient demographics, investigation, diagnosis, and management, particularly in relation to blood-thinning medications. Ethical approval for this study, conducted at West Suffolk Hospital, was obtained from the Ethics Committee of the hospital and the study was registered with the local audit committee (project number 5496) in January 2018. In consideration of the study design, which did not involve the collection of patient identification data, verbal consent was deemed appropriate and was approved by the Ethics Committee. All participants provided informed consent for the audit and subsequent publication of findings. To safeguard privacy and confidentiality, results were presented anonymously.

## Results

A total of 170 patients were admitted to the emergency department of West Suffolk Hospital during the study period. 93 (54.71%) patients were included in the study, and the remaining patients were excluded because of age or not being on blood thinning medications. The average age of the participants was 82 years and 58 (62.3%) were male. Seven patients (8%) presented with a shock index less than one and had CT angiograms that did not reveal any active bleeding. All patients were followed up for three months. These patients were resuscitated using blood transfusions. Five patients were taking warfarin, which was reversed using a vitamin K (Phytomenadione) antagonist and fresh frozen plasma with consultation from the haematologist, while two patients on clopidogrel were managed with platelet transfusions.

**Figure 1.**
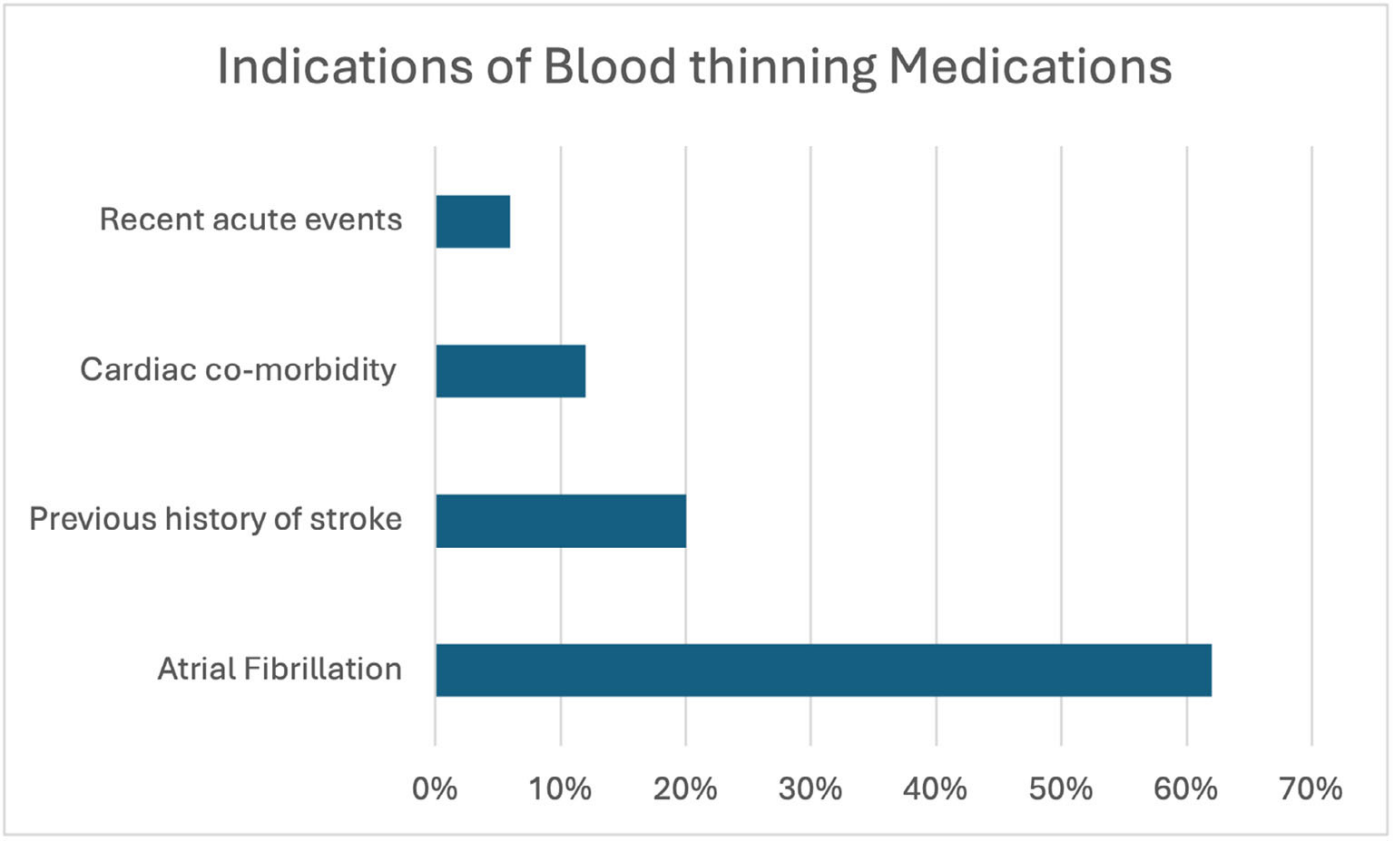
Graphical representation of indication of blood thinning medication in study population.

The distribution of indications for blood-thinning medications among the study population is illustrated in Figure 1. ^8^ Atrial fibrillation accounted for 59 (52%) patients, previous stroke accounted for 18 (20%), and cardiac comorbidities such as previous myocardial infarction accounted for 11(12%). Only five (6%) patients had a recent acute events: Cardiac co morbidity, cardiac intervention, deep vein thrombosis and pulmonary embolism within last 3 months within the last 3 months.

**Figure 2.**
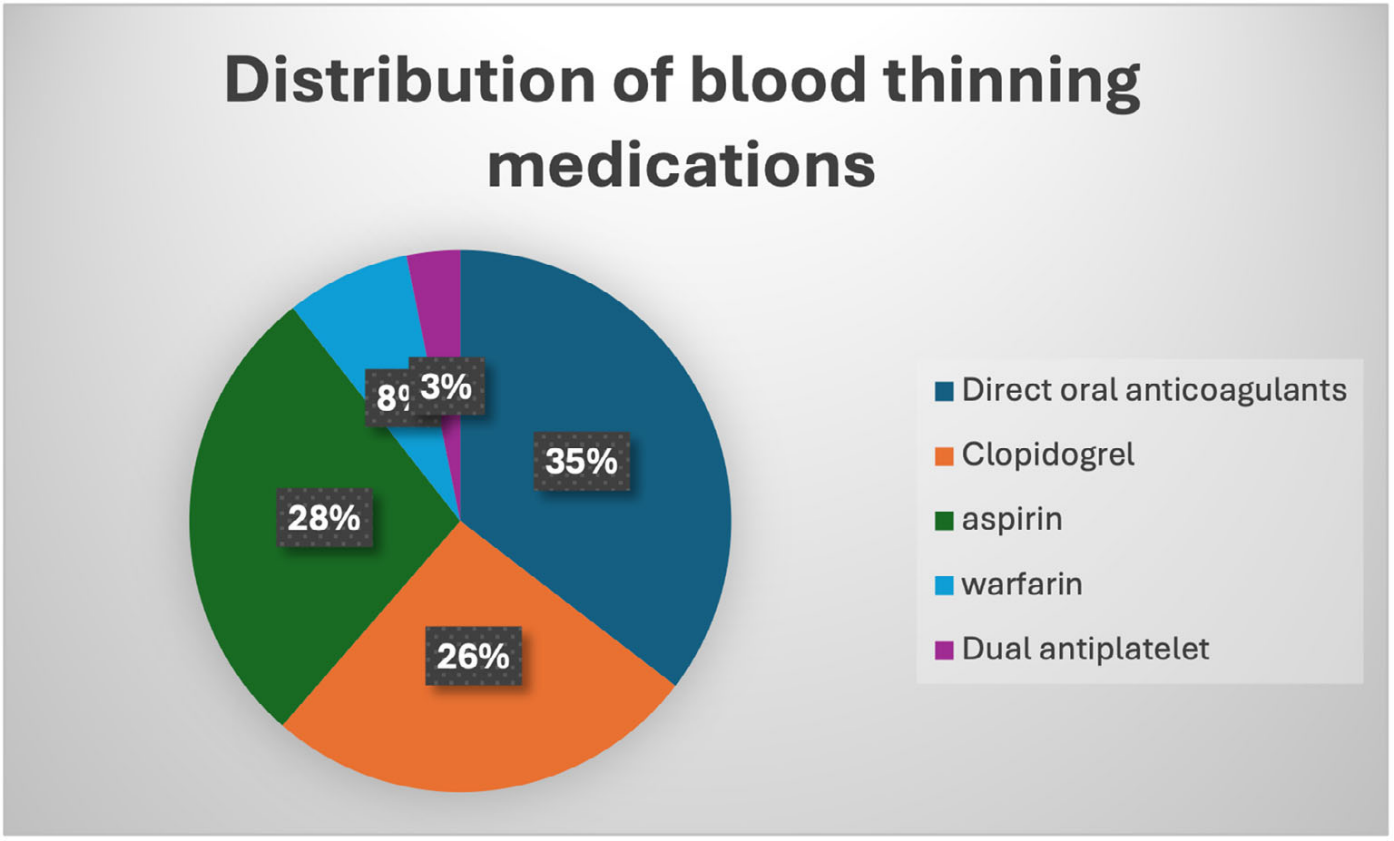
Illustration of the distribution of blood-thinning medications within the study population.

In the realm of anticoagulant therapy, 33 patients (35.4%) were administered direct oral anticoagulants (DOACs), while clopidogrel was prescribed for 16 patients (14.88%). Aspirin was the choice for 29 patients (32.5%), and 13 patients (14.2%) were on warfarin. Additionally, dual antiplatelet therapy was utilized by 2 patients (3.2%), as depicted in Figure 2. ^9^

**Figure 3.**
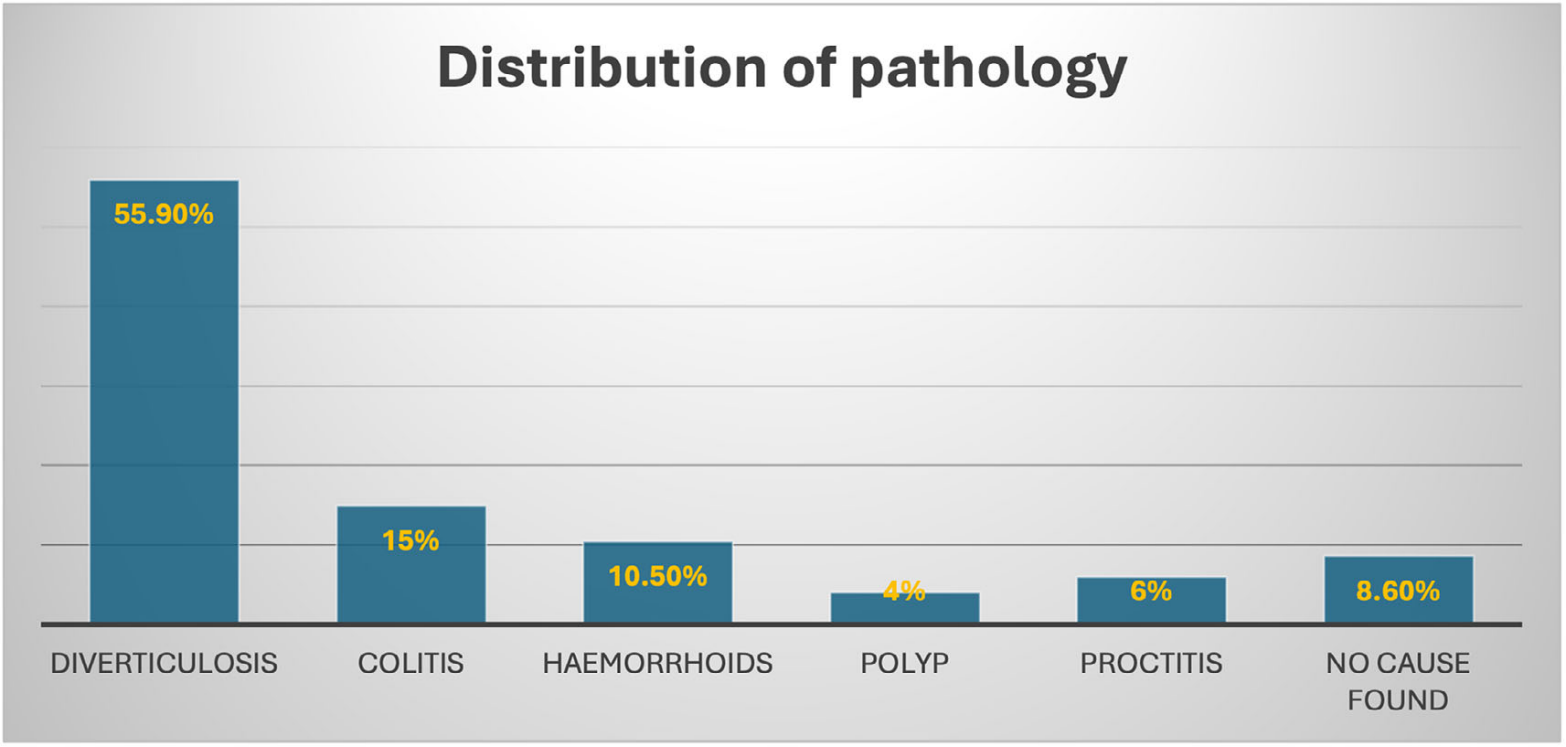
Illustration of the distribution of colorectal pathology among the study population.

During the same admission, patients underwent gastroscopy and flexible sigmoidoscopy to investigate the potential cause of rectal bleeding. Of the 97 patients, 52 (55.9%) were diagnosed with diverticulosis, 14 (15%) had acute colitis, and eight (8.60%) had no identified cause. Notably, none of the patients in this age group was diagnosed with malignancy. The distribution of the pathologies is provided in Figure 3. ^10^ No patient required emergency surgery or radiological intervention, as the bleeding ceased with conservative management.

In terms of restarting the medication, all patients had a prolonged conversation in terms of the risks and benefits of restarting the medication as well as consultation with medical colleagues when required. There was a clear demonstration of a lack of uniformity in terms of plans for restarting blood-thinning medications.

**Figure 4.**
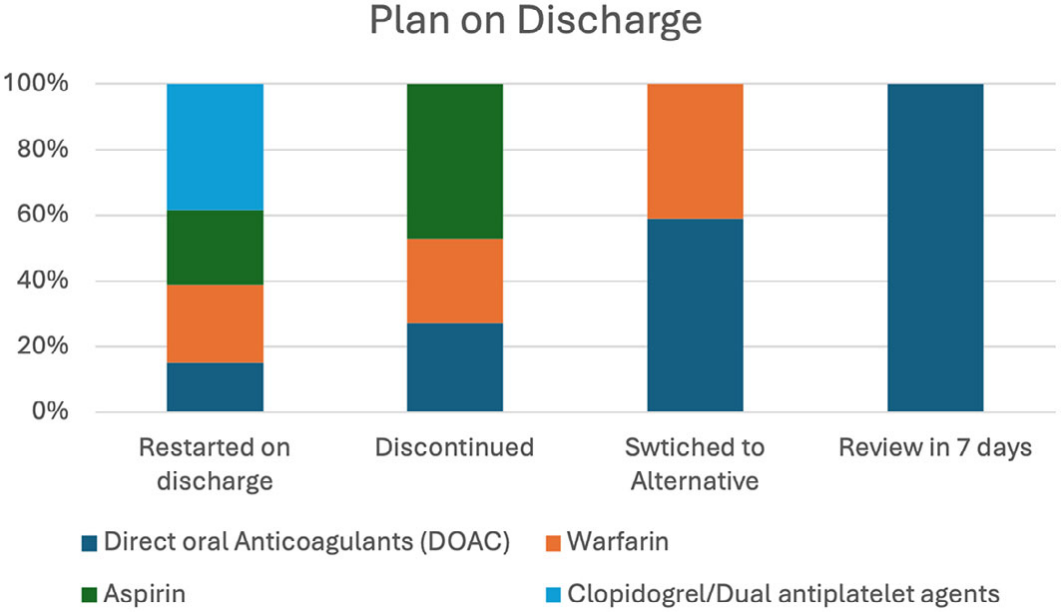
Illustration of the graphical representation of discharge advice regarding various blood-thinning medications among the study population.

The management of blood-thinning medications at discharge was meticulously tailored to individual patient needs. Among those prescribed DOACs, 14 patients (39%) were recommenced upon discharge, with 7 (23%) patients transitioning to alternative therapies such as warfarin, and a further 7 (23%) of them not resuming anticoagulation. Additionally, 5 (15%) patients were slated for delayed recommencement after seven days. Conversely, 8 (62%) patients on warfarin were reinstated upon discharge, while 3 (22%) of them ceased treatment and others were switched to direct oral anticoagulants. Notably, 18 (60%) patients of aspirin recipients were recommenced, while discontinuation was deemed appropriate for the remainder. However, all patients prescribed clopidogrel and dual antiplatelet therapy were promptly initiated on these agents upon discharge. This comprehensive management strategy is visualized in Figure 4, ^11^ exemplifying the nuanced approach to optimizing patient outcomes.

Patients were followed up for a period of 3 months, and readmission was mainly observed in patients on antiplatelet agents.

## Discussion

The aging population in the UK, coupled with an increasing number of patients being initiated on blood thinners, has led to a significant increase in patients presenting with rectal bleeding. This trend is particularly notable in the elderly population, with individuals over 60 years of age constituting a substantial proportion of those affected. ^12^ The incidence is higher in older patients and those taking multiple medications, and the incidence rate increases with age, with a greater than 200 -fold increase from the third to the ninth decades of life. ^13^ The management of blood thinners in elderly patients with rectal bleeding presents a challenge due to the limited existing literature and lack of uniform guidelines, particularly regarding the recommendation of medications upon discharge. Current practices for handling lower gastrointestinal bleeding are influenced by local expertise and service availability, given the scarcity of high-quality evidence guiding such cases. ^14^

The majority of elderly patients presenting with bleeding per rectum often consume blood thinners, which can have a significant impact on their management and outcomes due to the comorbidities that frequently require the use of multiple medications, including anticoagulants, to treat conditions such as atrial fibrillation and venous thromboembolism. ^15^ In our study, 54.71% of the patients were blood thinners, which was a significant number, with the majority being male. In a study by Kim et al., ^16^12% of patients with rectal bleeding required ICU admission; similarly, our study showed that 8% of patients presented with severe shock and were managed conservatively according to the protocol.

In terms of indications for blood-thinning medications in elderly patients, a study by Russo et al. ^17^ revealed that the most common indication is atrial fibrillation, followed by a history of stroke. Similarly, in our study, 52% of the patients had atrial fibrillation, followed by a history of stroke.

In the context of blood-thinning medications, a study by Afzal et al. ^18^ analysed the prescription trends of oral anticoagulants in England over a decade. This study highlighted the use of Direct Oral Anticoagulants (DOACs), such as apixaban and rivaroxaban, along with warfarin, in managing conditions such as atrial fibrillation. A review by Minichelli et al. ^19^ emphasized the significant impact of DOACs on anticoagulation therapy, providing a more convenient alternative to warfarin owing to factors such as reduced need for monitoring and fewer drug interactions, which is consistent with the finding that 35.4% of patients were prescribed DOACs in the context of blood-thinning medications. Lenz-Habijan et al. ^20^ highlighted the importance of dual antiplatelet treatment (DAPT) involving aspirin and clopidogrel in reducing thromboembolic complications. This is relevant as 14.88% of patients were on clopidogrel and 32.5% were taking aspirin in the context of blood-thinning medications.

Diverticulosis was the most common cause of lower GI bleeding in the very elderly population, accounting for 66.6% of cases. ^21^ Similarly, in our study, 60% of patients were diagnosed with diverticulosis. This aligns with the notion that, in a significant proportion of elderly patients with lower GI bleeding, no specific cause may be identified, with diverticulosis being a common finding.

Studies have shown that resuming anticoagulation therapy after a major bleeding event can induce anxiety for both clinicians and patients, as the decision to prevent thromboembolic events by restarting anticoagulation or reducing the risk of recurrent bleeding by discontinuing anticoagulation is challenging. ^22^ The decision-making process often involves a multidisciplinary approach to ensure the best patient outcomes. While restarting oral anticoagulation alone has been associated with an increased risk of major bleeding compared to non-resumption of treatment, there is a difference in the risk of recurrent gastrointestinal bleeding between patients who restarted antithrombotic treatment and those who did not. This highlights the complexity of the decision-making process and the need for individualized treatment strategies. ^23^ In our study, all patients on dual antiplatelet agents and the majority of patients on aspirin were reintroduced at the time of discharge. In terms of patients on DOAC, 39% of them were restarted, 23% switched to an alternative, and 5% stopped the medication completely; in terms of warfarin, the majority of them were restarted as soon as possible.

## Conclusion

The management of blood-thinning drugs in patients with rectal bleeding is subject to individual patient variations, and a pragmatic approach is desired. This necessitates larger trials to achieve greater standardization.

### Ethics and consent

Ethical approval for this study, conducted at West Suffolk Hospital, was obtained from the Ethics Committee of the hospital and the study was registered with the local audit committee (project number 5496) in January 2018. In consideration of the study design, which did not involve the collection of patient identification data, verbal consent was deemed appropriate and was approved by the Ethics Committee. All participants provided informed consent for the audit and subsequent publication of findings. To safeguard privacy and confidentiality, results were presented anonymously.

## Data Availability

Underlying data Figshare: Management of Blood thinning medications in Elderly populations presenting with Rectal bleeding: Are we doing right?
• Fig. 1: Graphical representation of indication of blood thinning medication in study population. (Version 1). figshare. https://doi.org/10.6084/m9.figshare.25705563.v1. ^8^ The Project contains following data: Atrial fibrillation was seen in 52% (n=59), previous stroke in 20% (n=18), and cardiac comorbidities, including myocardial infarction, in 12% (n=11). Recent acute events occurred in only 6% (n=5) within the last three months. Data are available under the terms of the Creative Commons Attribution 4.0 International license (CC-BY 4.0). Figshare: Management of Blood thinning medications in Elderly populations presenting with Rectal bleeding: Are we doing right?
• Fig. 2: Illustration of the distribution of blood-thinning medications within the study population. (Version 1). figshare. https://doi.org/10.6084/m9.figshare.25705614.v1. ^9^ The Project contains following data: Among the anticoagulant therapies, DOACs were administered to 35.4% (n=33) of patients, while 14.88% (n=16) received clopidogrel. Aspirin was prescribed for 32.5% (n=29) of patients, and warfarin for 14.2% (n=13). Dual antiplatelet therapy was used by 3.2% (n=2) of patients. Data are available under the terms of the Creative Commons Attribution 4.0 International license (CC-BY 4.0). Figshare: Management of Blood thinning medications in Elderly populations presenting with Rectal bleeding: Are we doing right?
• Fig. 3: Illustration of the distribution of colorectal pathology among the study population. (Version 2). figshare. https://doi.org/10.6084/m9.figshare.25705638.v2. ^10^ The Project contains following data: During admission, 55.9% (n=52) were diagnosed with diverticulosis, 15% (n=14) with acute colitis, and 8.60% (n=8) had no identified cause for rectal bleeding. No malignancies were detected in this age group. Data are available under the terms of the Creative Commons Attribution 4.0 International license (CC-BY 4.0). Figshare: Management of Blood thinning medications in Elderly populations presenting with Rectal bleeding: Are we doing right?
• Fig. 4: Illustration of the graphical representation of discharge advice regarding various blood-thinning medications among the study population (Version 1). figshare. https://doi.org/10.6084/m9.figshare.25705653.v1. ^11^ The Project contains following data: The figure shows discharge management of blood-thinning medications: 39% of DOAC users resumed, 23% switched to warfarin, and 15% planned delayed restart. For warfarin, 62% resumed, 22% ceased, and others transitioned. Aspirin was recommenced in 60%, while clopidogrel and dual therapy were initiated universally. Data are available under the terms of the Creative Commons Attribution 4.0 International license (CC-BY 4.0).
