# Supplementary material for: Management of blood thinning medications in elderly populations presenting with rectal bleeding: Are we doing right?
Source: F1000Res. 2024 Jun 4;13:569. [Version 1] doi: 10.12688/f1000research.149548.1 (PMC11208849; doi:10.12688/f1000research.149548.1)
Supplement: Peer review history

# Reviewer report: Reviewer response for version 1

**DOI:** 10.5256/f1000research.164026.r289819

**Published:** 2024-06-26

**Authors:** prakash krishnasamy

**Affiliations:** 1 Sri Ramakrishna hospital, coimbatore, India

This study addresses  on the day-to-day emergency clinical scenario that every health centre faces and very much relevant in everyday practice. It clearly depicts its data on the above question with relevant citations . Its results are much in concordance with  day to day practice . This study has made a genuine effort in trying to study the current practices in the management of such patients.

Is the case presented with sufficient detail to be useful for teaching or other practitioners?

Yes

Is the work clearly and accurately presented and does it cite the current literature?

Yes

If applicable, is the statistical analysis and its interpretation appropriate?

Yes

Are all the source data underlying the results available to ensure full reproducibility?

Yes

Are the conclusions drawn adequately supported by the results?

Yes

Is the background of the case’s history and progression described in sufficient detail?

Yes

Reviewer Expertise:

clinical management of similar group of patients included in the study

I confirm that I have read this submission and believe that I have an appropriate level of expertise to confirm that it is of an acceptable scientific standard.

# Reviewer report: Reviewer response for version 1

**DOI:** 10.5256/f1000research.164026.r289816

**Published:** 2024-06-21

**Authors:** Mohana Raj Thanpal

**Affiliations:** 1 Department of General Surgery, Kuala Lambur hospital, Kuala Lambur, kuala, Malaysia

The topic that was chosen is appropriate as managing this group of patients is complicated. This article clearly demonstrates the heterogeneity in management of these group of patients. 

 The presentation of the study is clear and straightforward.

 The results and figures are presented in easily understandable way. 

 The discussion is also well structured.

Is the case presented with sufficient detail to be useful for teaching or other practitioners?

Yes

Is the work clearly and accurately presented and does it cite the current literature?

Yes

If applicable, is the statistical analysis and its interpretation appropriate?

Not applicable

Are all the source data underlying the results available to ensure full reproducibility?

Yes

Are the conclusions drawn adequately supported by the results?

Yes

Is the background of the case’s history and progression described in sufficient detail?

Yes

Reviewer Expertise:

colorectal surgery

I confirm that I have read this submission and believe that I have an appropriate level of expertise to confirm that it is of an acceptable scientific standard.
